# Music Augmented With Isochronic Auditory Beats or Vibrotactile Stimulation Does Not Affect Subsequent Ergometer Cycling Performance: A Pilot Study

**DOI:** 10.3389/fnhum.2021.713193

**Published:** 2021-09-13

**Authors:** Adam Fry, Stephen Braren, Nicholas Pitaro, Brandon Larson, David Putrino

**Affiliations:** ^1^Department of Rehabilitation and Human Performance, Icahn School of Medicine at Mount Sinai, New York, NY, United States; ^2^Department of Applied Psychology, New York University, New York, NY, United States; ^3^Red Bull High Performance, Red Bull North America, Santa Monica, CA, United States

**Keywords:** augmented music, exercise, isochronic tones, sport, vibration

## Abstract

Methods to enhance the ergogenic effects of music are of interest to athletes of all abilities. The aim of this pilot study was to investigate the ergogenic effects of two commercially available methods of music augmentation: auditory beats and vibrotactile stimulation. Six male and five female cyclists/triathletes cycled for 7 minutes at three different intensities: a rate of perceived exertion (RPE) of 11 (“light”), RPE of 15 (“hard”), and a 7-minute time-trial. Before each 7-minute bout of cycling, participants listened to 10 minutes of self-selected music (MUS), or the same music with the addition of either isochronic auditory beats (ABS) or vibrotactile stimulation via SUBPAC^TM^ (VIB). MUS, ABS and VIB trials were performed in a randomized order. Power output was measured during cycling and felt arousal and feeling scores were recorded at timepoints throughout the protocol. The results found the augmented MUS interventions did not influence power output with no significant main effect of trial (*p* = 0.44, η^2^ = 0.09) or trial × cycling intensity interaction (*p* = 0.11, η^2^ = 0.20). Similarly, both felt arousal and feeling scores were unchanged between the MUS, ABS, and VIB trials (*p* > 0.05). In conclusion, this pilot study indicated an ineffectiveness of the ABS and VIB to affect subsequent 7-min cycling performance compared to self-selected MUS alone.

## Introduction

Music trial (MUS) is a commonly adopted accompaniment to exercise and sport. Athletes of diverse ability levels use MUS before or during exercise to enhance performance, motivation, and enjoyment in their training. Listening to MUS can improve endurance performance (Atkinson et al., 2004), explosive power production (Biagini et al., 2012) and skill-based performance (Alrashid, 2015). MUS may help optimize an athlete’s state of arousal for exercise, which can confer performance gains (Karageorghis and Priest, 2012). Alternatively, MUS can improve mood and increase the pleasure derived from exercising (Terry et al., 2020). Methods of augmenting MUS to enhance its positive psychophysiological effects are therefore of interest as a possible pathway to improvements in either exercise performance or the experience of exercise.

Auditory beats trial (ABS) and vibration trial (VIB) are two methods of MUS augmentation that have gained popularity among consumers. Spotify playlists and YouTube videos featuring ABS have received millions of listens/views. This media has been promoted as accompaniments for a variety of activities including meditation, yoga, sleep improvement, studying, and stress relief. Likewise, commercially available VIB devices, such as the SUBPAC^TM^, have gained popularity among consumers aiming to enhance the subjective experience of sound or MUS. The accessibility of these novel technologies has made them of interest to athletes, especially those already using MUS in their practice. It is therefore important to investigate the ergogenic effects of these technologies to inform both athletes and coaches.

Auditory beat stimulation is a method of cortical entrainment whereby auditory stimuli are used to induce a neuro-oscillatory response that follows the frequency band of the stimulation (Chaieb et al., 2015). Auditory beat stimulation has been applied to modulate arousal; an important psychophysiological determinant of sports performance (Yerkes and Dodson, 1908). For example, ABS at low (theta/alpha) frequencies have been found to increase relaxation (Le Scouarnec et al., 2001), whereas ABS at higher (beta/gamma) frequencies may heighten arousal (Lane et al., 1998; Colzato et al., 2015). Beta/gamma frequency ABS have previously been found to enhance performance on cognitive tasks (Lane et al., 1998; Colzato et al., 2015; Shekar et al., 2018). Methods of auditory beat stimulation include isochronic tones, binaural beats, and monaural beats. Isochronic tones involve a pure tone that is turned on and off a fixed number of times per second to create an auditory beat at that frequency (Engelbregt et al., 2019). Isochronic tones can be overlaid onto MUS, however, the potential to modulate the psychophysiological effects of MUS via the addition of isochronic tones is unknown.

Vibrotactile stimulation applied to the skin, is a potent activator of the sensorimotor system. Commercially available devices provide the opportunity to synchronously pair VIB with MUS. When paired, vibrotactile and auditory stimuli may be synergized within the brain (Foxe et al., 2002; Schürmann et al., 2006). However, whether this combination of vibrotactile and auditory stimuli might augment the ergogenic effects of MUS is unknown.

In practice, MUS is often impractical during sports participation or banned during competition. Instead, individuals often listen to MUS prior to sports participation as a priming tool (Loizou and Karageorghis, 2015). Previous investigations have indicated increases in power output during ergometer cycling tasks following 10 min of MUS listening compared to no MUS prior to the task (Eliakim et al., 2007; Jarraya et al., 2012). Therefore, the goal of this exploratory pilot study was to test the hypothesis that listening to 10 min of MUS overlaid with isochronic tones or VIB prior to ergometer cycling would increase power output compared to listening to the same MUS alone.

## Materials and Methods

### Participants

Eleven endurance trained cyclists and triathletes participated in this study. These were six males (mean ± standard deviation, age: 25 ± 8 years; mass: 76.0 ± 8.5 kg; VO_2_max: 52.7 ± 8.6 ml⋅kg^–1^⋅min^–1^) and five females (age: 22 ± 3 years; mass: 55.4 ± 2.5 kg; VO_2_max: 43.0 ± 3.6 ml⋅kg^–1^⋅min^–1^). All participants were in active cycling training during their off-season (males: 192 ± 111 km⋅week^–1^; females: 94 ± 30 km⋅week^–1^). Each participant provided written informed consent prior to the study, which was approved by the Icahn School of Medicine institutional review board (IRB-18-00780). We recruited athletes who were highly familiar with cycling exercise to reduce the variance in cycling performance not attributable to the intervention (e.g., learning effects or muscle soreness).

### Study Design

The study involved a randomized cross-over design, including five visits to the laboratory within a 10-day timespan. All study visits were completed at the same time of day and were separated by 48 or 72 h. Participants were requested to abstain from strenuous exercise between study visits, consume 500 ml of water 1 h before the start of each session, and keep the final meal before each session consistent in both content and timing.

### Visit 1

The first visit served to establish VO_2_max. Following a warm-up, participants performed a continuous incremental cycling test to volitional exhaustion on a cycling ergometer (SRM indoor trainer, Jülich, Germany). Initial power output was set to 80 W for females and 120 W for males for 60 s, before increasing by 5 W every 15 s. Participants continued cycling until they were unable to maintain their freely chosen cadence; denoted by a drop of 10 rpm. Pulmonary gas exchange was assessed breath-by-breath throughout the test using a calibrated metabolic cart (Vmax Vyntus CPX, Vyaire Medical, Mettawa, IL, United States). VO_2_max was defined as the highest 30-s VO_2_ during the test. After a 15-min cool down, participants were familiarized with each of the three MUS conditions: MUS alone, MUS plus isochronic ABS and MUS plus VIB, using a 40-s piece of demo MUS.

### Visits 2–5

**FIGURE 1:**
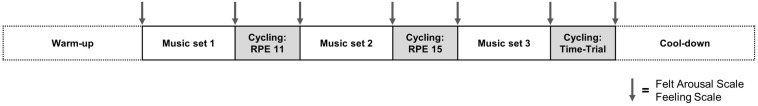
The study protocol for visits 2–5. Visit 2 (familiarization) involved no music (MUS), whereas visits 3–5 included MUS alone (control trial), MUS plus isochronic auditory beat stimulation, or MUS plus vibrotactile stimulation (VIB), and were performed in a randomized order. Each MUS set was 10 min, and each bout of cycling was 7 min.

The protocol for visits 2–5 is illustrated in Figure 1. These visits were identical except for the MUS intervention. Visit 2 served as a familiarization of the cycling tasks to minimize any potential learning effects on subsequent cycling performances, particularly the 7-min time-trial. This visit did not include any MUS listening. On visits 3–5, before each bout of cycling participants listened to either 10 min of MUS alone or the same MUS with the addition of either isochronic ABS or VIB. The MUS, ABS, and VIB trials were completed in a randomized order. The MUS trial served as the control trial against which the two augmented MUS trials were compared.

Participants completed the same 15-min warm-up on each visit, which consisted of 7 min cycling at a rate of perceived exertion of 9 (RPE; Borg, 1970), 2 min at RPE 11, 2 min at RPE 13, 1-min at RPE 15, and 3 min at RPE 11. Following this warm-up, participants listened to their first 10-min MUS track (set 1 – see MUS) before remounting the ergometer to cycle for 7 min at an RPE of 11 – “Light.” Participants then listened to their second 10-min MUS track (set 2) before cycling for 7 min at an RPE of 15 – “Hard” and listened to their third 10-min MUS track (set 3) before completing a 7-min time-trial. The time-trial began at a rolling cadence of 50 rpm, and participants were instructed to cycle as far as possible in 7 min. At the end of the time-trial, participants remained on the ergometer for a 15-min cool-down.

Throughout all the cycling in visits 2–5, participants were able to manually adjust their gear and alter their cadence continuously. A stop-clock displaying elapsed time was displayed. All other performance metrics such as power, gear, and cadence were hidden from the participants view. No verbal or motivational feedback was provided to the participants during the protocol. Water was provided for the participants to drink ad libitum.

Responses to the Felt Arousal Scale (Svebak and Murgatroyd, 1985) and Feeling Scale (Hardy and Rejeski, 1989) were recorded immediately after each of the following: the warm up, MUS set 1, cycling at RPE 11, MUS set 2, cycling at RPE 15, MUS set 3, and the time-trial. The Felt Arousal Scale is a six-point scale from 1 (“Low Arousal”) to 6 (“High Arousal”) and the Feeling Scale is an eleven-point scale from +5 (“Very Good”) to −5 (“Very bad”).

### Music

The MUS participants listened to during the study visits was self-selected prior to their first visit. Participants were asked to select a total of nine MUS tracks; three tracks they would choose to listen to before cycling at a “light intensity”, three tracks they would choose to listen to before cycling at a “hard intensity”, and three tracks they would choose to listen to before cycling at a “maximal intensity (i.e., a time-trial).” Each set of three tracks were concatenated into 10-min tracks, and are referred to here as MUS sets 1, 2, and 3, respectively. This was achieved by editing the tracks for duration including removing any dead space during transitions and then leveling to create a single track in MP3 format.

For the ABS condition, a 40 Hz isochronic tone was added to the same MUS from the MUS condition. Isochronic tones are generated using a single tone that is rapidly turned on and off to create a distinct beat. Our isochronic tone involved a 440 Hz pure tone (sinusoidal waveform) played every 0.025 s with a 50% duty cycle to create a distinct 40 Hz beat (Supplementary Figure 1). A 40 Hz auditory beat was chosen as it may be optimal for increasing gamma band cortical activity (Ross et al., 2014). A 440 Hz pure tone was chosen as 440 Hz is a widely used tuning standard for western MUS. The amplitude of the 440 Hz pure tone was modulated with a minimal on/off ramp. Audio leveling reduced the amplitude of the isochronic tones to −14 to −16 dB below the base MUS’s highest dB level and a one second fade in and fade out was applied. This dB level was chosen to allow the isochronic tone to be clearly audible without overpowering the MUS throughout the dynamic range of the base MUS. The −2 dB window (−14 to −16 dB) provided leeway between concatenated MUS tracks where the base audio was louder or softer and was set to accommodate for comfort while listening. All MUS editing was performed in advance of the laboratory visits using Garage Band software.

For the VIB condition, participants wore a VIB backpack (Subpac M2X, Toronto, ON, Canada) while listening to the same MUS from the MUS condition. This backpack uses sound transducing actuators to transform auditory inputs within the 1–200 Hz frequency band (colloquially, the bass line of the MUS) into VIB applied to the user’s torso. The MUS was inputted to the backpack via a standard 3.5-mm auxiliary cable and forwarded to headphones for synchronous presentation of the audio and VIB. The MUS drove the backpack’s output such that increases in MUS volume also increased the amplitude of the vibration. Participants had continuous control of the amplitude of vibration using a gain dial on the device, which did not impact audio volume.

For each of the three MUS conditions, participants had continuous control of the MUS volume. Participants listened to their MUS using an MP3 player (Apple iPod Touch 6th Ed., Cupertino, CA, United States) and headphones (Bose Soundlink II, Framingham, MA, United States) in a private adjoining room that provided both a comfortable chair and open floor space. During visit 2, participants entered this same room for 10-min without MUS.

### Statistical Analyses

The goal of our statistical analyses was to explore the effect of the augmented MUS experiences on cycling performance and self-reported feeling/arousal. We used repeated measures analysis of covariance (ANCOVA) to test for differences in power, cadence, arousal, and feeling while accounting for each participant’s gender, age, body mass, VO_2_ max, and average weekly cycling distance. Specifically, for power and cadence we used a series of 3 × 3, trial (MUS, ABS, and VIB) × cycling intensity (light, hard, and time-trial) ANCOVAs. For arousal and feeling scores, we subtracted the score given at the end of warm-up from all subsequent scores and this change score was used as the dependent measure for analyses. Subsequently, we applied 3 × 3 × 2, trial × cycling intensity × activity (post-MUS listening/pre-cycling or immediately post-cycling) ANCOVAs. Additionally, one 2 × 3, trial × cycling intensity ANCOVA was conducted to compare mean power output between the MUS trial and the familiarization trial, which involved no MUS. Eta squared values were used to provide effect sizes.

Covariates that did not contribute significantly to a model were removed from the final model to preserve degrees of freedom. Mauchly’s test of sphericity was evaluated for each model and *p*-values were adjusted using the Greenhouse-Geisser correction where appropriate. Finally, the false discovery rate method was used to control for multiple comparisons where main effects were significant. All statistical analyses were conducted using SPSS version 25.

As no previous publication regarding augmented MUS vs. MUS alone on power output during subsequent cycling was available to derive an effect size for power analyses, the sample size for this study was based on two previous studies that investigated the effects of MUS vs. no MUS on power output during subsequent cycling performance (Eliakim et al., 2007; Jarraya et al., 2012). One of these studies found a significant effect with 12 participants (Jarraya et al., 2012) and the other study included a larger sample of 24 participants from which a power analysis (effect size: *d* = 1.33; α = 0.05; power = 0.9) indicated that only 9 participants may have been required to determine significant effects. An initial sample of 12 participants was therefore considered appropriate for this exploratory pilot study. A further power analysis using the results of this study would then determine the sample size required to appropriately power an analysis of augmented MUS vs. MUS alone on power output during subsequent cycling. This was performed after 11 participants due to a pause in recruitment/testing. The results of this analysis (see section “Results”) revealed a very large number of participants would be required to find a significant effect. This was considered impractical, and the study was therefore terminated at 11 participants. All power analyses were performed using G^∗^Power version 3.1 (Faul et al., 2007).

## Results

### Cycling Performance

**FIGURE 2:**
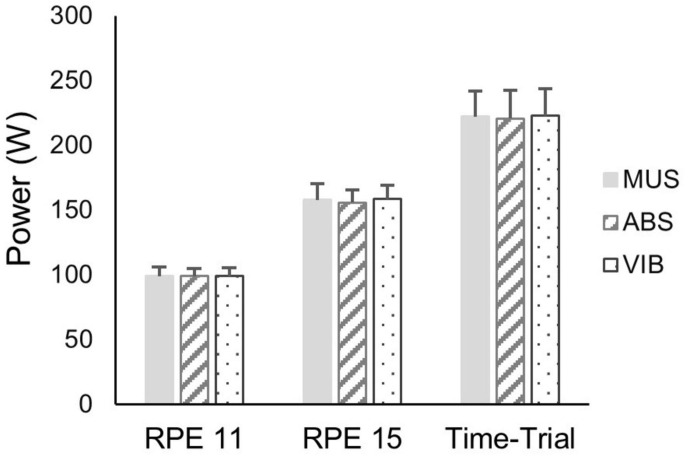
Mean power during the 7 min of light intensity cycling (RPE = 11), hard intensity cycling (RPE = 15) and during the 7-min time-trial across each of the three trials: MUS alone, MUS plus isochronic auditory beat stimulation (ABS), and MUS plus VIB. Values are mean ± standard error. No significant effect of trial or trial × cycling intensity interaction was identified.

The average power output produced across the trials is shown in Figure 2. As expected, power output significantly increased between the three cycling intensities; light, hard, and time-trial (ANCOVA: *F*_(2,18)_ = 12.18, *p <* 0.01, η^2^ = 0.60; *t*-tests: light vs. hard: *t*_(10)_ = −8.30, *p <* 0.01, hard vs. time-trial: *t*_(10)_ = −5.04, *p <* 0.01). However, there was no main effect of trial (MUS vs. ABS vs. VIB) on mean power output (*F*_(2,18)_ = 0.86, *p* = 0.44, η^2^ = 0.09), and no significant trial × cycling intensity interaction effect was identified (*F*_(4,18)_ = 2.02, *p* = 0.11, η^2^ = 0.20) indicating there were no cycling intensity dependent differences between the trials. The post-hoc power analysis indicated that 132 participants would be required to achieve a statistically significant main effect of trial on mean power output.

Mean power output in the first 30 s of each bout of cycling was not different between trials (*F*_(2,18)_ = 0.11, *p* = 0.89, η^2^ = 0.01) and no trial × cycling intensity interaction was identified (*F*_(4,18)_ = 0.89, *p* = 0.47, η^2^ = 0.10) (Table 1). There was also no significant main effect of trial on mean cadence (*F*_(2,18)_ = 3.08, *p* = 0.08, η^2^ = 0.28) or trial × cycling intensity interaction (*F*_(4,18)_ = 1.97, *p* = 0.12, η^2^ = 0.19) (Table 1).

**TABLE 1:** Mean cadence during the 7-min bouts of cycling and mean power output during the first 30 s.

		**MUS**	**ABS**	**VIB**
Cadence (rpm)	Light	85 ± 4	84 ± 4	86 ± 3
	Hard	94 ± 4	94 ± 5	95 ± 4
	Time-trial	102 ± 4	102 ± 5	102 ± 4
30-s power (W)	Light	93 ± 6	95 ± 6	93 ± 6
	Hard	134 ± 8	138 ± 7	140 ± 7
	Time-trial	205 ± 14	201 ± 17	211 ± 13

*Data are shown for each of the three trials: music alone (MUS), MUS plus isochronic auditory beat stimulation (ABS), and MUS plus vibrotactile stimulation (VIB). Values are mean ± standard error.*

### Arousal and Feeling

**FIGURE 3:**
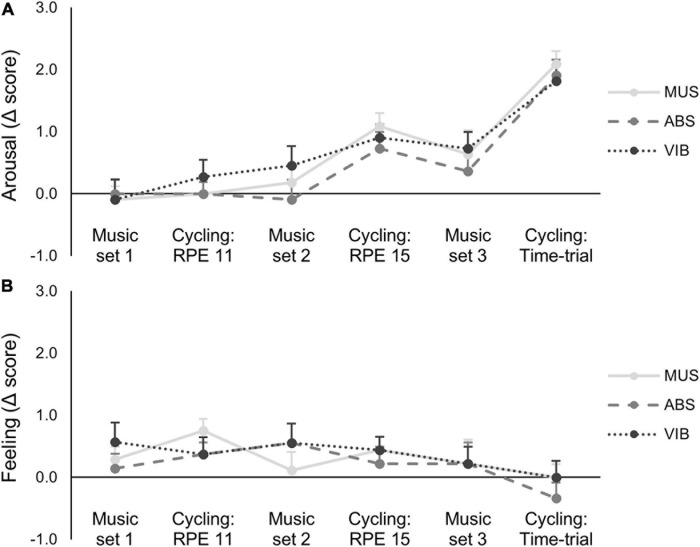
Change in felt arousal **(A)** and feeling scores **(B)** across each of the three trials: MUS alone, MUS plus isochronic auditory beat stimulation (ABS), and MUS plus VIB. Data are change in self-reported scores from warm-up (mean ± standard error). No significant effects of trial or trial × cycling intensity interactions were identified.

Felt arousal scores changed significantly during the trials (Figure 3A). Significant differences were found within both the three post-MUS-listening scores (*F*_(2,20)_ = 5.42, *p* = 0.04, η^2^ = 0.37; light vs. hard: *p* = 0.06; light vs. time-trial: *p* = 0.01; hard vs. time-trial: *p <* 0.01) and the three post-cycling scores (*F*_(2,20)_ = 23.47, *p <* 0.01, η^2^ = 0.72; light vs. hard: *p <* 0.01; light vs. time-trial: *p <* 0.001; hard vs. time-trial: *p* < 0.001). Post-cycling arousal scores were also higher than the post-MUS-listening scores (*F*_(2,20)_ = 12.23, *p* < 0.01, η^2^ = 0.57). However, there was no main effect of trial (MUS vs. ABS vs. VIB) on either post-MUS-listening (*F*_(2,20)_ = 1.30, *p* = 0.29, η^2^ = 0.13) or post-cycling arousal scores (*F*_(2,20)_ = 0.34, *p* = 0.69, η^2^ = 0.04), and the trial × cycling intensity interactions were not significant for either post-MUS-listening (*F*_(4,40)_ = 2.16, *p* = 0.10, η^2^ = 0.19) or post-cycling arousal scores (*F*_(4,40)_ = 0.80, *p* = 0.53, η^2^ = 0.08). Unlike arousal, self-reported feeling scores were unchanged throughout the trials (Figure 3B) with no significant difference within either the post- MUS-listening scores (*F*_(2,16)_ = 0.90, *p* = 0.43, η^2^ = 0.11) or the post-cycling scores (*F*_(2,16)_ = 0.07, *p* = 0.93, η^2^ = 0.01), and no difference between the post-MUS-listening and post-cycling scores (*F*_(2,20)_ = 1.85, *p* = 0.21, η^2^ = 0.21). Similarly, no main effect of trial on either the post-MUS-listening (*F*_(2,16)_ = 1.61, *p* = 0.23, η^2^ = 0.19) or post-cycling feeling scores was found (*F*_(2,16)_ = 0.27, *p* = 0.76, η^2^ = 0.04), and no significant trial × cycling intensity interaction was identified for either post-MUS-listening (*F*_(4,32)_ = 2.23, *p* = 0.13, η^2^ = 0.24) or post-cycling arousal scores (*F*_(4,32)_ = 0.22, *p* = 0.77, η^2^ = 0.03).

### Music Alone

Power output during the MUS trial was not different to power output during the familiarization trial. There was no main effect of trial (MUS vs. familiarization) on mean power output (*F*_(1,9)_ = 2.99, *p* = 0.12, η^2^ = 0.27), and no significant trial × cycling intensity interaction effect (*F*_(2,18)_ = 0.30, *p* = 0.74, η^2^ = 0.04).

## Discussion

This exploratory pilot study investigated the ergogenic effects of two methods of MUS augmentation: isochronic ABS and VIB. The main finding was that listening to MUS with the addition of either isochronic ABS or VIB did not affect subsequent cycling performance or self-reported feeling and arousal when compared to the self-selected MUS alone.

Mean power output was consistent between the MUS, ABS, and VIB trials. Cycling cadence was also unaffected, indicating that the kinematics by which participants achieved these power outputs was unchanged. In one previous study, improvements in 10-km cycling performance with MUS listening during exercise resulted from a faster opening 3 km (Atkinson et al., 2004). The authors suggested that MUS might have higher ergogenic effects at the onset of maximal cycling or in shorter maximal cycling bouts. We therefore examined the mean power output during the first 30 s of the time-trial. This was also consistent between trials, indicating there were no short-lasting effects of the augmented MUS at the start of the time-trial.

Neither the “light”, “hard” or maximal cycling was impacted by the augmented MUS listening. This is in contrast to two studies that found an increase in mean power output during submaximal cycling at a fixed RPE with MUS compared to no MUS (Carlier et al., 2017) as well as lower RPE during cycling at a fixed submaximal intensity (Potteiger et al., 2000), however, both these studies listened to MUS during cycling rather than prior to cycling. Two other studies found increases in power output during a maximal cycling task following 10 min of MUS listening compared to no MUS prior to cycling (Eliakim et al., 2007; Jarraya et al., 2012), however, these studies involved 30-s Wingate tasks compared to a 7-min time-trial effort as per the current study. Moreover, participants also listened to the MUS while cycling in the warm-up (Eliakim et al., 2007; Jarraya et al., 2012). It is therefore possible that the MUS increased Wingate performance indirectly by augmenting the efficacy of the warm-up.

In addition to power output, this study also examined felt arousal and feeling throughout the experiment. Self-reported arousal increased with cycling intensity and was higher following the bouts of cycling than following the MUS listening (immediately prior to beginning the cycling), however, neither the isochronic ABS nor VIB had any significant effect on the arousal scores. Previous studies have shown MUS to be a strong modulator of arousal, which can improve exercise performance (Priest and Karageorghis, 2008). Although listening to more stimulating MUS before a maximal exercise task may confer greater psychophysiological adaptations than less stimulating MUS (Karageorghis et al., 1996), the addition of isochronic ABS or VIB to self-selected MUS did not alter felt arousal in the current study. Contrary to previous studies that have found significant effects of MUS and exercise on subjective feeling (Karageorghis and Priest, 2008), self-reported feeling scores were unchanged throughout the trials and were similar between the MUS, ABS and VIB trials. This indicated that neither the MUS, isochronic ABS, VIB or 7-min cycling efforts affected the self-reported feeling of the current participants.

Previous research on high frequency ABS (≥13 Hz) has produced multiple reports of improved performance on cognitive and simple motor tasks. For example, high frequency binaural ABS were shown to elicit improved performance on a vigilance task where participants were instructed to rapidly press a computer key when a target was displayed on the screen (Lane et al., 1998). This study also showed that the high frequency binaural ABS ameliorated the negative effects on mood of a monotonous task and reduced task-related mental fatigue compared to both binaural ABS in the low frequency range and a pure tone. Binaural ABS at 40 Hz have also been found to improve reaction time via an increase in attention and arousal (Shekar et al., 2018). These studies imply high frequency ABS may be able to modulate psychophysiological states, which could potentially be applied to sports or exercise performance. However, findings are equivocal, and ABS have often failed to produce changes in physiological biomarkers including heart rate, blood pressure, skin conductance response, or even the targeted electrocortical activity (Carter, 2008; López-Caballero and Escera, 2017). The results of the current study suggest ABS may not be effective in changing psychophysiology pertinent to subsequent cycling performance.

Synchronous auditory and VIB may produce a supra-additive cortical response; meaning simultaneous stimulation with these two modalities results in a larger response than that predicted by the sum of the unimodal responses (Foxe et al., 2002; Schürmann et al., 2006). This phenomenon may alter the subjective intensity of MUS when it is presented alongside synchronous tactile stimulation. For example, perceived loudness may be increased (Gillmeister and Eimer, 2007). To date, however, there is little evidence that this sensory integration leads to an augmented ergogenic effect.

There are several limitations to this study that should be considered. Firstly, this exploratory pilot study included a small sample of 11 participants. As described in the section “Methods” section, the decision not to pursue a larger number of participants was based upon a power analysis performed using the results of the current 11 participants, which indicated that over one hundred participants would be required to identify a statistically significant main effect of the MUS augmentation modalities on cycling power output. Recruiting these participants was considered impractical and, in the authors’ opinion, unlikely to lead to a substantial change in effect size, which was negligible (η^2^ = 0.09). Nevertheless, we feel it is important to disseminate these non-significant pilot results as they might be of interest to athletes and coaches and to prevent repetition of research efforts from other scientists interested in this field.

Secondly, no difference in mean power output was observed between the MUS trial and the familiarization trial, which was performed without any MUS. Although order effects should be considered in comparisons with the familiarization trial, this result indicates that MUS alone did not have an ergogenic effect on subsequent cycling performance within the current study design. Our ability to determine whether the isochronic ABS or the VIB augmented an ergogenic effect of MUS alone was therefore limited. Nevertheless, the results still demonstrate that 10 min of isochronic ABS and VIB did not have an ergogenic effect on the subsequent cycling exercise. The current study design included MUS listening prior to the 7-min cycling efforts owing to bans and impracticalities of MUS listening during many sports. However, one previous review suggested MUS prior to exercise may benefit performance in shorter, predominantly anaerobic tasks but found insufficient evidence to support a performance enhancing effect of MUS before predominantly aerobic exercise (Smirmaul, 2017). It is therefore impossible to exclude the possibility that either isochronic ABS or VIB might augment the ergogenic effects of MUS in paradigms where such effects are present.

Thirdly, the MUS characteristics that might be most effectively combined with auditory beat stimulation or VIB is not yet known. While this is worthy of investigation, the purpose of the current study was to objectively assess two emerging technologies (*already being used by early adopters*) in an ecologically valid manner (*with self-selected music*). Moreover, prescribing specific MUS to exercisers also has limitations; previous studies have demonstrated that MUS preference can have a significant impact on the ergogenic effect of that MUS (Nakamura et al., 2010; Karow et al., 2020).

Fourthly, while participants were required to keep their final meal before each session consistent in both content and timing, we did not mandate precise replication of caloric intake or collect food diaries to control for inter-trial differences in macronutrient distribution. Given power outputs were highly consistent between trials, it does not seem likely that small differences in diet may have influenced the overall findings of this study. This supposition is supported by previous studies that have demonstrated carbohydrate intake prior to short cycling time-trials (∼33 min or less) does not affect performance (Palmer et al., 1998; Paul et al., 2003; Jeukendrup et al., 2008).

In conclusion, the results of this small exploratory pilot study (11 participants) indicate an ineffectiveness of the isochronic ABS and VIB to affect subsequent 7-min exercise performance, compared to self-selected MUS alone. These results are pertinent to athletes and coaches interested in maximizing the effects of MUS in their training or prior to competition. Future research might consider the potential ergogenic effects of listening to augmented MUS during exercise performance.

## Data Availability Statement

The raw data supporting the conclusions of this article will be made available by the authors, without undue reservation.

## Ethics Statement

The studies involving human participants were reviewed and approved by the Icahn School of Medicine Institutional Review Board. The patients/participants provided their written informed consent to participate in this study.

## Author Contributions

DP and BL were responsible for the study design. AF performed the data collection. SB performed the statistical analyses. AF, NP, and SB drafted the manuscript with editing from DP and BL.

## Conflict of Interest

BL was employed by Red Bull North America. The authors declare that this study received funding from Red Bull North America. The funder was not involved in the study design, collection, analysis, interpretation of data, the writing of this article or the decision to submit it for publication.

## Publisher’s Note

All claims expressed in this article are solely those of the authors and do not necessarily represent those of their affiliated organizations, or those of the publisher, the editors and the reviewers. Any product that may be evaluated in this article, or claim that may be made by its manufacturer, is not guaranteed or endorsed by the publisher.

## Supplementary Material

Supplementary Figure 1 The 40 Hz isochronic tone **(A)** and an illustration of the mixing of the music with the isochronic tone **(B)**.
