# Supplementary figures and images for: Music Augmented With Isochronic Auditory Beats or Vibrotactile Stimulation Does Not Affect Subsequent Ergometer Cycling Performance: A Pilot Study
Source: Front Hum Neurosci. 2021 Sep 13;15:713193. doi: 10.3389/fnhum.2021.713193 (PMC8475787; doi:10.3389/fnhum.2021.713193)

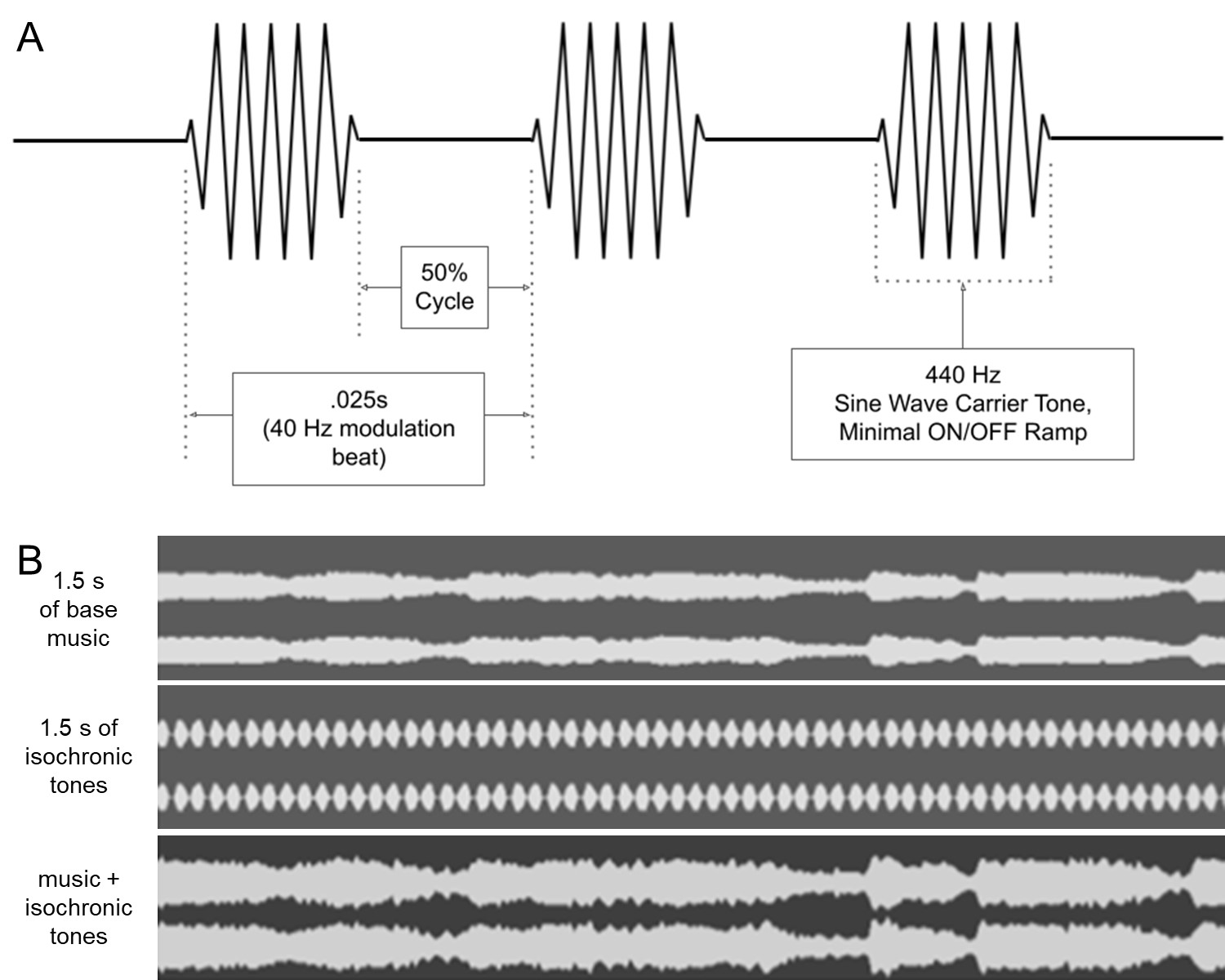

Supplement: Supplementary Figure 1 — The 40 Hz isochronic tone (A) and an illustration of the mixing of the music with the isochronic tone (B). [file Image_1.JPEG]
